# A novel technique of B2–B3 single-puncture bridging through liver parenchyma enabling endoscopic ultrasound-guided hepaticogastrostomy for rescue drainage

**DOI:** 10.1055/a-2663-8418

**Published:** 2025-08-20

**Authors:** Hiroki Koda, Kazuo Hara, Nozomi Okuno, Shin Haba, Takamichi Kuwahara, Shimpei Matsumoto, Tomoki Ogata

**Affiliations:** 1 538357 Department of Gastroenterology, Aichi Cancer Center, Nagoya, Japan

Malignant hilar biliary strictures often result in separation of intrahepatic bile ducts, necessitating placement of multiple stents to achieve adequate drainage 1 . Endoscopic ultrasound-guided hepaticogastrostomy (EUS-HGS) has emerged as a valuable salvage option when transpapillary access is limited 2 3 . Although EUS-guided bridging techniques have been reported, intrahepatic bridging through the liver parenchyma has not yet been described 4 .

A 62-year-old man with pancreatic tail cancer and peritoneal dissemination presented with malignant hilar biliary obstruction. Four transpapillary biliary stents had previously been placed. However, the patient developed focal cholangitis with septic shock due to undrained B2 and B3 bile ducts ( Fig. 1 ). EUS-HGS was considered, as additional transpapillary drainage was not feasible.

**Fig. 1:**
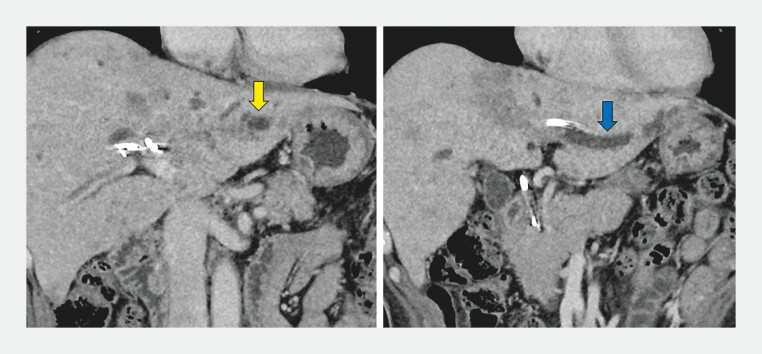
CT image demonstrating cholangitis in the undrained lateral segment despite placement of four stents for malignant hilar biliary obstruction. The B2 (yellow arrow) and B3 (blue arrow) ducts are isolated and noncommunicating.

Linear EUS (EG740UT; FUJIFILM) clearly identified both the B2 and B3 ducts. Due to their proximity to the stricture and the inevitable need for transesophageal puncture, direct EUS-HGS to B2 was not feasible. As B2 and B3 were aligned linearly and both required drainage, a bridging technique was selected. A single transhepatic puncture was made using a 19G needle (EZ Shot 3 Plus; Olympus), traversing the liver parenchyma from B2 to B3 ( Fig. 2 ). A 0.025-inch guidewire was advanced, followed by deployment of an uncovered self-expandable metal stent (ZEOSTENT V, 8 mm × 6 cm; ZEON MEDICAL) to bridge the two ducts ( Video 1 , Fig. 2 , Fig. 3 ).

B2–B3 bridging through liver parenchyma combined with EUS-HGS enabled drainage of the lateral segment inaccessible by conventional endoscopic approach.Video 1

**Fig. 2:**
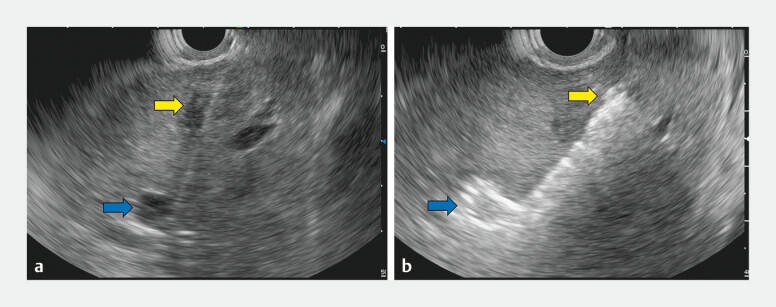
Under EUS guidance, a single puncture was used to traverse B2 (yellow arrow) and subsequently access B3 (blue arrow), achieving transhepatic bridging between B2 and B3. **a** Needle puncture of B2 and B3. **b** Bridging between B2 and B3.

**Fig. 3:**
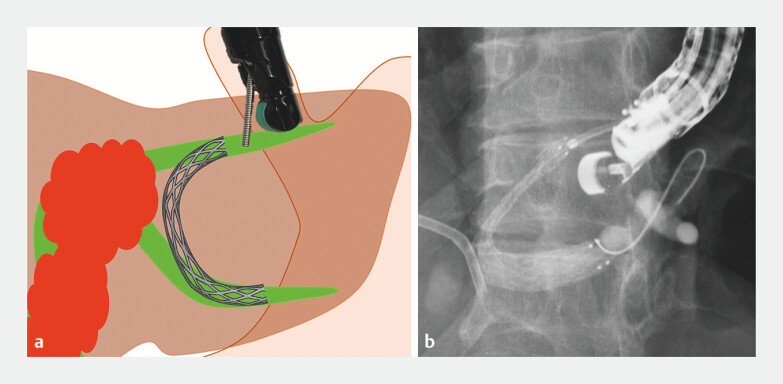
The stent bridges the ducts through the liver parenchyma, rather than within the stenotic bile duct. **a** Schematic illustration. **b** Fluoroscopic image.

**Fig. 4:**
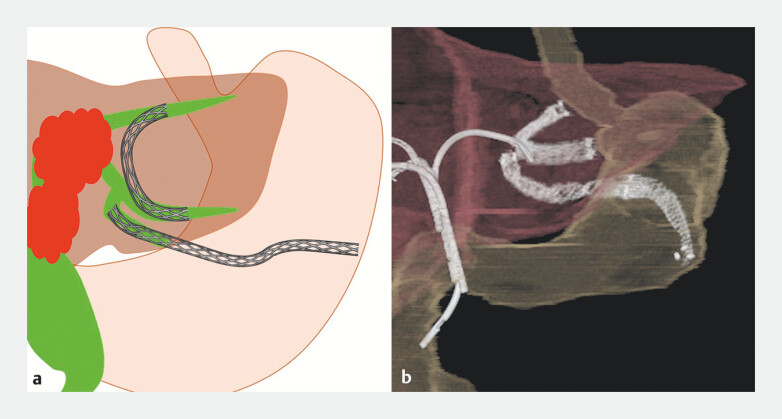
Post-procedural positional relationship of the EUS-guided biliary drainage stent. **a** Schematic illustration. **b** Reconstructed CT image.

Subsequently, EUS-HGS was performed via a separate puncture into B3 using a fully covered metal stent (HANAROSTENT Biliary Benefit, 6 mm × 12 cm) ( Fig. 4 ). Follow-up CT imaging confirmed effective biliary decompression. The patient’s cholangitis resolved promptly, and chemotherapy was resumed.

This case highlights the technical feasibility and clinical value of single-puncture intrahepatic bridging from B2 to B3 by directly traversing the liver parenchyma, enabling effective EUS-HGS and rescue drainage of the lateral hepatic segment.

Endoscopy_UCTN_Code_TTT_1AS_2AH

## Notes

Endoscopy E-Videos https://eref.thieme.de/e-videos
*E-Videos* is an open access online section of the journal *Endoscopy* , reporting on interesting cases and new techniques in gastroenterological endoscopy. All papers include a high-quality video and are published with a Creative Commons CC-BY license. Endoscopy E-Videos qualify for HINARI discounts and waivers and eligibility is automatically checked during the submission process. We grant 100% waivers to articles whose corresponding authors are based in Group A countries and 50% waivers to those who are based in Group B countries as classified by Research4Life (see: https://www.research4life.org/access/eligibility/ ). This section has its own submission website at https://mc.manuscriptcentral.com/e-videos .
